# A comparative analysis of the nutrient and phytochemical richness among different varieties of quinoa in China

**DOI:** 10.1002/fsn3.4113

**Published:** 2024-04-24

**Authors:** Yuan‐Mou Tang, Yi‐Zhi Liu, Yan‐Hong Zhang, Ya‐Nan Cao, Pan‐Pan Song, Li‐Ming Hou, Lian‐Xin Peng

**Affiliations:** ^1^ Key Laboratory of Coarse Cereal Processing of Ministry of Agriculture and Rural Affairs Chengdu University Chengdu China; ^2^ Affiliated Hospital of Chengdu University of Chinese Medicine Chengdu Sichuan China

**Keywords:** bioactive compounds, germplasm diversity, nutritional composition, quality evaluation, quinoa (*Chenopodium quinoa*)

## Abstract

Quinoa is a nutrient‐dense pseudocereal that has garnered global attention for its potential to bolster food security and nutrition. Despite its celebrated status, the detailed nutritional profiles of various quinoa varieties remain poorly understood, which poses a significant barrier to the strategic cultivation and utilization of quinoa's genetic diversity to combat malnutrition. The impetus for this research lies in the urgent need to identify superior quinoa strains that can be tailored to meet specific nutritional requirements and adapt to diverse agro‐ecological zones. Our findings reveal substantial variation in nutrient content across different quinoa varieties, highlighting the variety ZLZX‐8 as a particularly nutrient‐rich strain with the highest levels of protein, fat, essential fatty acids, amino acids, and key minerals such as Mg, K, and Zn. Moreover, ZLZX‐8's exceptional antioxidant capacity suggests it may have additional health benefits beyond its macronutrient profile. In contrast, ZLZX‐7 stands out for its dietary fiber and phenolic content, which are critical for digestive health and disease prevention, respectively. Meanwhile, ZLZX‐5, with its high starch content, could be better suited for energy production in dietary applications. Notably, the study also uncovers a correlation between grain color and nutrient profile, with colored quinoa varieties exhibiting superior fiber, inositol, phenolic content, and antioxidant activity compared to their white counterparts. This work lays the groundwork for an informed selection of quinoa varieties that can enhance dietary quality, support local and global food systems, and contribute to the fight against malnutrition.

## Graphical abstract

Systematic comparison of the nutritional and active ingredients of different varieties of quinoa. The antioxidant activity of different varieties of quinoa was compared. The quality of quinoa of different colors was analyzed.

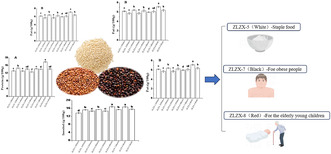

## 1 INTRODUCTION

Quinoa (*Chenopodium quinoa* Willd.) is a pseudocereal of the Amaranthaceae genus (James, 2009). It is an annual plant with twin cotyledons, and there are currently about 250 varieties of quinoa (Navruz‐Varli & Sanlier, 2016). Quinoa seeds are mostly oval, with different grain color characteristics such as white, purple, red, black, and yellow (Wang et al., 2016). Quinoa is the only monomeric plant that can meet the basic nutritional needs of the human body (Ocampo et al., 2023), it is referred to as “nutritional gold,” “super grain,” and “mother of food” by international nutritionists. Quinoa grain has a high concentration of amino acids, fiber, minerals, vitamins, saponins, and phenolics that can help alleviate various biological diseases in the human body (Chen et al., 2023). Quinoa exhibits a diverse range of applications, including antioxidant properties, anti‐cancer potentiality, anti‐inflammatory effects, as well as hypoglycemic and lipid‐lowering abilities. Moreover, it demonstrates promising prospects in weight management. Consequently, quinoa holds the potential to enhance the overall nutritional status of populations while also serving as a preventive measure against various diseases (Jan et al., 2023; Navruz‐Varli & Sanlier, 2016; Ren et al., 2023).

Due to genetic variations, the nutritional and active ingredient content of different quinoa varieties also varies. Therefore, it is essential to select the appropriate specialized variety based on the distribution characteristics of quinoa's nutritional and functional components during food processing. Consequently, a systematic analysis of the quality of diverse quinoa varieties becomes imperative. In earlier studies, scholars have already conducted analyses on the primary nutritional components across various quinoa varieties. For instance, Chen and Liao (2020) conducted a study on the nutritional composition of seven varieties of quinoa and found that Taiqi black quinoa is rich in protein, high in total dietary fiber, and low in fat, making it particularly suitable for weight‐loss purposes. Taiqi White Quinoa and Shangri‐La Red Quinoa exhibited superior essential amino acid content scores, rendering them more appropriate for infants and young children. On the other hand, Shangri‐La Black Chenoa demonstrated a high level of potassium and a low level of sodium, making it a suitable choice for middle‐aged and elderly individuals. Chen et al. (2023) provide a detailed evaluation of the abundant nutrients of quinoa seeds from thirty varieties with different colors and different origins, including soluble protein, soluble sugar, amino acids, vitamins, fatty acids, and saponin. Despite numerous reports on the quality analysis of different quinoa varieties as a significant grain resource, there remains a lack of comparability and systematic evaluation due to the utilization of scattered indicators in analysis and testing, particularly within the same place of origin.

The present study investigated nine different quinoa varieties as the subjects of research, systematically analyzing and evaluating their main nutrients, fatty acid content, amino acid content, mineral elements, inositol, phenolic components, and antioxidant activities. These findings provide data support for understanding the quality variations among different quinoa varieties and establish a theoretical foundation for the development of differentiated quinoa products.

## 2 MATERIALS AND METHODS

### 2.1 Materials and reagents

The quinoa samples, namely ZLZX‐1 (white grain), ZLZX‐2 (white grain), ZLZX‐3 (white grain), ZLZX‐4 (white grain), ZLZX‐5 (white grain), ZLZX‐6 (black grain), ZLZX‐7 (black grain), ZLZX‐8 (red grain), and ZLZX‐9 (red grain), obtained from Jintang County in Chengdu and provided by the Key Laboratory of Coarse Cereal Processing, Ministry of Agriculture and Rural Affairs at Chengdu University, were utilized for this study. These samples, identified as *Chenopodium quinoa* Willd. in the amaranth family by Professor Zhao Gang, were ground and passed through a 60‐mesh sieve.

2,2‐Diaza‐bis (3‐ethyl‐benzothiazole‐6‐sulfonic acid) diammonium salt (ABTS) was procured from Shanghai Ampu Experimental Technology Co., Ltd.; 1,1‐Diphenyl‐2‐trinitrophenylhydrazine (DPPH) from Sigma Aldrich Trading Co., Ltd.; Mg, K, Ca, Mn, Fe, Cu, Zn, and Na element mixed standard solution from the Chinese Academy of Metrology; tryptophan standard from Shanghai Ampu Experimental Technology Co., Ltd.; 17 amino acid standard solutions from a German company SYKAM; and 37 kinds of fatty acid methyl ester mixed standard solution from Shanghai Ampu Experimental Technology Co., Ltd. All other chemicals and solvents used in the study are analytical grade.

### 2.2 Determination of the nutritional content of quinoa

The analytical procedures for assessing the nutritional components of quinoa were meticulously adapted from established methods to ensure accuracy and reliability. The detection of quinoa protein was conducted using the AOAC Kjeldahl method with appropriate modifications (Grappin & Horwitz, 1988). Fat determination was performed through Soxhlet extraction, following the method of Patel et al. (2009) with appropriate modifications. Starch determination was carried out according to the method of Friedemann and Witt (1967) with appropriate modifications. Dietary fiber analysis followed the method of McCleary et al. (2010) with appropriate modifications. Fatty acid profiling was performed based on the procedure described by Golay et al. (2009) with appropriate modifications. Amino acid quantification was conducted following the approach outlined by Elkin and Griffith (1985) with appropriate modifications. Mineral element detection adhered to the methodology proposed by Pacquette et al. (2018) with appropriate modifications.

### 2.3 Amino acid score

The amino acid content of each variety of quinoa was converted into milligrams of amino acids per gram of protein, and subsequently, the nutritional value assessment was conducted based on the FAO/WHO best ratio model. The amino acid score (AAS) and chemical score (CS) were then derived using the following formula:

AAS = [amino acid content in sample protein (mg/g)]/[corresponding essential amino acid content in FAO/WHO scoring standard model (mg/g)];

CS = [Amino acid content in sample protein (mg/g)]/[Egg corresponding essential amino acid content (mg/g)].

### 2.4 Determination of inositol

The inositol content in quinoa was determined using gas chromatography, a method established by the research group during the initial phase (Zhang et al., 2021). The sample weighing 2.00 g should be placed in a 25‐mL volumetric flask. Then, add two‐thirds of the volume of a 70% ethanol solution to the flask and perform ultrasound extraction for 30 min. After that, adjust the volume to the scale with 70% ethanol and mix thoroughly before filtering. Finally, transfer 5 mL of the filtered solution into a rotary evaporator (RE‐52AA; Shanghai Yarong Biochemical Instrument Factory). Add 5 mL of absolute ethanol to the evaporation flask, followed by an additional 5 mL of absolute ethanol for continuous spinning and evaporation until complete dryness is achieved. Subsequently, add 4 mL of silanization reagent to the evaporation flask and dissolve it on a mixer using vortexing. Place the mixture in an 80°C water bath for a derivatization reaction with a duration of 20 min. After completion, remove it from the water bath and allow it to cool down to room temperature. Add 10 mL of water and vortex thoroughly before adding 5 mL of n‐hexane for extraction through vortexing for a period of 2 min. Transfer the resulting solution into a centrifuge tube and centrifuge at a speed of 8000 r/min for 5 min. Collect the supernatant for determination using gas chromatography (Agilent Technologies Co., Ltd., Agilent7890B).

The HP‐5 capillary column (30 m × 0.32 mm, 0.25 μm) was employed with a nitrogen flow rate of 1.0 mL/min. The inlet temperature was set at 280°C, while the FID (flame ionization detector) temperature was maintained at 300°C. A volume of 1.0 μL was injected using the split injection mode with a split ratio of 10:1. The temperature program consisted of holding at 120°C for 2 min, followed by an increase in temperature at a rate of 10°C/min until reaching 250°C and holding for another 5 min, then further increasing the temperature at a rate of 30°C/min to reach and holding at 300°C for an additional 5 min.

### 2.5 Determination of polyphenolic compounds

Appropriate adjustments were made to the method of determining quinoa polyphenolic compounds in the early stages of the research group (Ma et al., 2016; Wei et al., 2018). Accurately weigh 1.00 g of quinoa (ground and passed through a 60‐mesh sieve) in a 50‐mL Erlenmeyer flask. Add two‐thirds of the volume of a 90% methanol aqueous solution and perform ultrasound‐assisted extraction in a water bath at 50°C for 30 min. Cool the mixture to room temperature, adjust the volume to scale using a 90% methanol aqueous solution, vigorously shake, transfer 10 mL of the solution into a 50 mL centrifuge tube, and centrifuge at 11179 *g* for 5 min. Collect the supernatant by passing it through an organic filter membrane with a pore size of 0.22 μm. The measurement was performed on a rapid‐resolution liquid chromatography system (ACQUITY UPLC I‐Class, Waters, USA).

The ACQUITY UPLC® BEHC18 column (2.1 mm × 50 mm, 1.7 μm) was used for analysis. The mobile phase consisted of methanol (A) and 0.2% aqueous glacial acetic acid (B). A gradient elution procedure was employed with the following conditions: 10–25% A from 0 to 3 min, followed by a linear increase to 40% A from 3 to 15 min, maintaining at 40% A from 15 to18 min, and finally decreasing back to10% A from 18 to 20 min. The detection wavelength was set at 247 nm and the injection volume was kept at 1.0 μL. The column temperature was maintained at 30°C while the flow rate was set at 0.2 mL/min.

Linear regression analysis using the concentration of each phenolic component as the x‐axis and the corresponding peak area as the y‐axis allowed the determination of the phenolic content in quinoa.

### 2.6 ABTS•+ scavenging activity assay

The methodology employed in this study was adapted from Peng et al. (2015) with slight modifications. A total of 1 mL of quinoa phenol extract was taken and placed into a 25 mL colorimetric tube. Subsequently, 4 mL of ABTS radical working solution was added to the tube and thoroughly mixed. After allowing the reaction to proceed for 30 min at room temperature under light‐protected conditions, the absorbance was measured at a wavelength of 734 nm. This determination process was repeated three times for accuracy. To establish a standard curve, various concentrations of Trolox were used as controls to determine absorbance values. The concentration of Trolox served as the x‐axis, while its corresponding absorbance value acted as the y‐axis on the graph plot. The free radical scavenging capacity of quinophenol extract against ABTS+• was then calculated using the established standard curve, with results reported in Trolox equivalent units (μmol/100 g) per 100 g of dry extract.

### 2.7 DPPH radical scavenging activity assay

The methodology employed in this study was adapted from Peng et al. (2015) with slight modifications. Take 2 mL of polyphenol extract and add it to 2 mL of a 0.1 mmol/L DPPH• methanol solution. Allow the reaction to occur at room temperature, protected from light, for a duration of 30 min. Measure the absorbance at a wavelength of 517 nm and repeat this process three times. To establish a standard curve, use different concentrations of Trolox as controls and plot the absorbance values against corresponding Trolox concentrations on an xy graph. Calculate the DPPH• radical scavenging capacity based on this standard curve, expressing the results as Trolox equivalent (μmol Trolox/100 g) per 100 g of dry extract.

### 2.8 Statistical analysis

All compound content is expressed by dry weight. All quality index results are averaged over three measurements. The *t*‐test was analyzed using IBM SPSS 19.0 (IBM Corporation, USA); the histogram was drawn using Origin 8.0 (OriginLab, Inc., USA) and the principal component analysis was analyzed using SIMCA‐P 11.0.

## 3 RESULTS AND ANALYSIS

### 3.1 Nutritional content of various quinoa varieties

**FIGURE 1:**
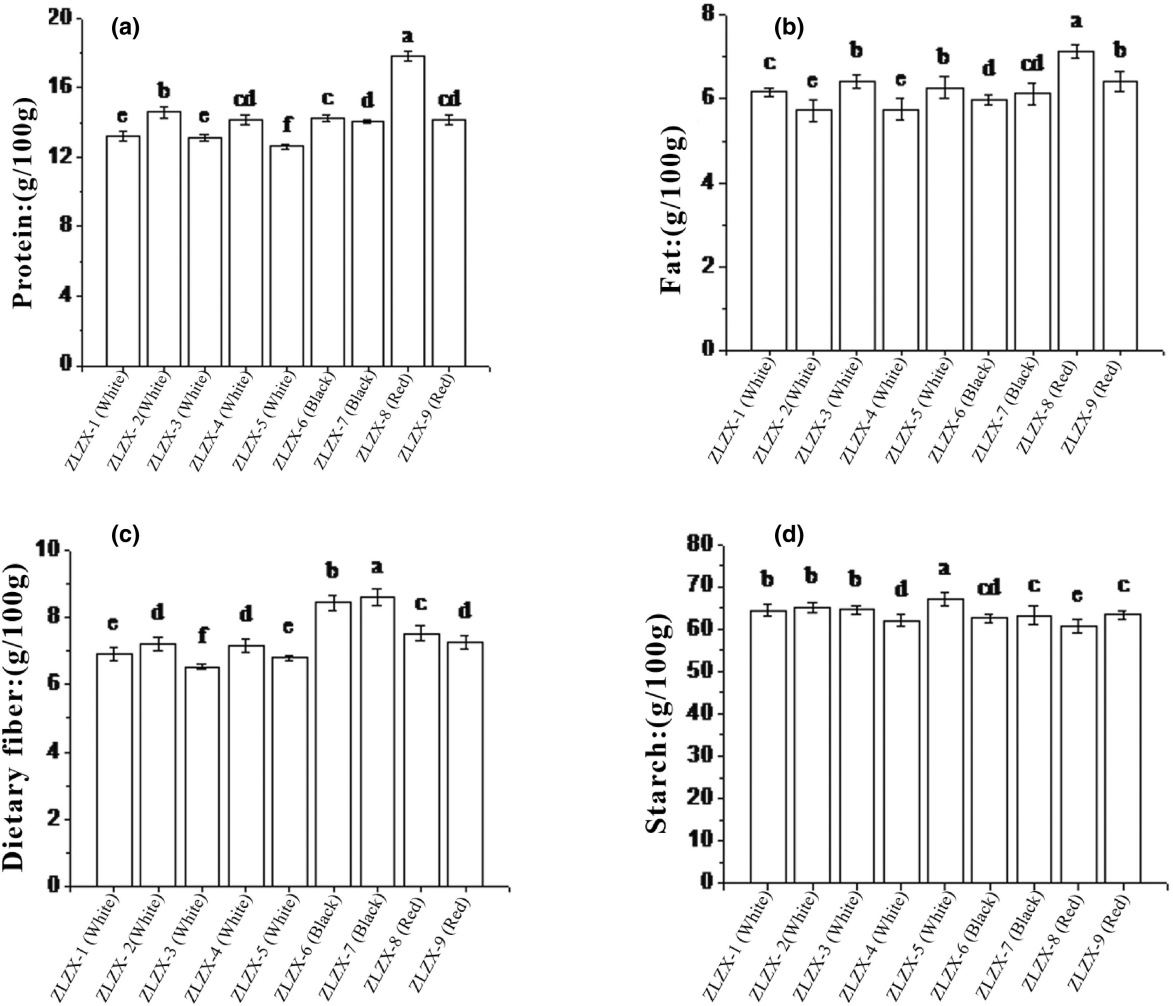
Nutritional general nutritional content of 9 varieties of quinoa. (a) protein content; (b) fat content; (c) dietary fiber content; (d) starch content; different lowercase letters are indicated for comparison at the 5% significance level.

The protein, fat, dietary fiber, and starch contents of the nine different varieties of quinoa are presented in Figure 1. Protein is an important factor in determining the quality of cereals. The protein content of quinoa could range from 11% to 19%, which is higher than millet (Saleh et al., 2013). Wheat (Kumar et al., 2011), black rice (Ito & Lacerda, 2019), buckwheat (Luthar et al., 2021), and oats are comparable (Sangwan et al., 2014). In Chen et al.'s (2023) study, the protein content of 30 varieties of quinoa was measured, which ranged from 11.4% to 19%. In our study, the protein content ranged from 12.61 to 17.77 g/100 g across the nine quinoa varieties. This may be because the seed resources come from the same origin, so the difference in protein content will be smaller. Among them, ZLZX‐8 (red grain) had the highest protein content and was significantly higher than that of other varieties (*p* < .05), and ZLZX‐5 (white grain) had the lowest protein content. The fat content is 5.79–7.16 g/100 g. Quinoa contains many high‐quality fatty acids, so the fat content is also significantly higher than other grains, similar to oats (Sangwan et al., 2014). Among them, ZLZX‐8 (red grain) had the highest fat content and was significantly higher than that of other varieties (*p* < .05). There was no significant difference in fat content between ZLZX‐3 (white grain), ZLZX‐9 (red grain), and ZLZX‐5 (white grain), and ZLZX‐2 (white grain) had the lowest fat content (*p* < .05). Comprehensive analysis showed that red quinoa had the highest fat content, followed by black quinoa, and white quinoa had the lowest. The dietary fiber content ranged from 6.53 to 8.61 g/100 g, with ZLZX‐7 (black grain) exhibiting the highest dietary fiber content, which was significantly higher than that of other varieties (*p <* .05). ZLZX‐6 (black grain), ZLZX‐8 (red grain), and ZLZX‐9 (red grain) followed suit, showing significant differences in content compared to each other (*p* < .05), and white quinoa displayed the lowest dietary fiber content. The results showed that the dietary fiber content was significantly correlated with quinoa color. The starch content ranges from 60.73 to 67.00 g/100 g, which surpasses the reported quinoa starch content in the U.S. National Nutrition Database (USDA, 2013). This may be related to the processing method of quinoa samples. The content of ZLZX‐5 (white grain) exhibited the highest levels, significantly surpassing those of other varieties (*p* < .05). Conversely, ZLZX‐8 (red grain) displayed the lowest starch content. Generally, white quinoa varieties exhibited significantly higher starch content than colored ones. Moreover, the nutritional composition of nine different quinoa varieties was similar to that reported by Rubén Vilcacundo et al. (2017).

### 3.2 Fatty acid composition of various quinoa varieties

**FIGURE 2:**
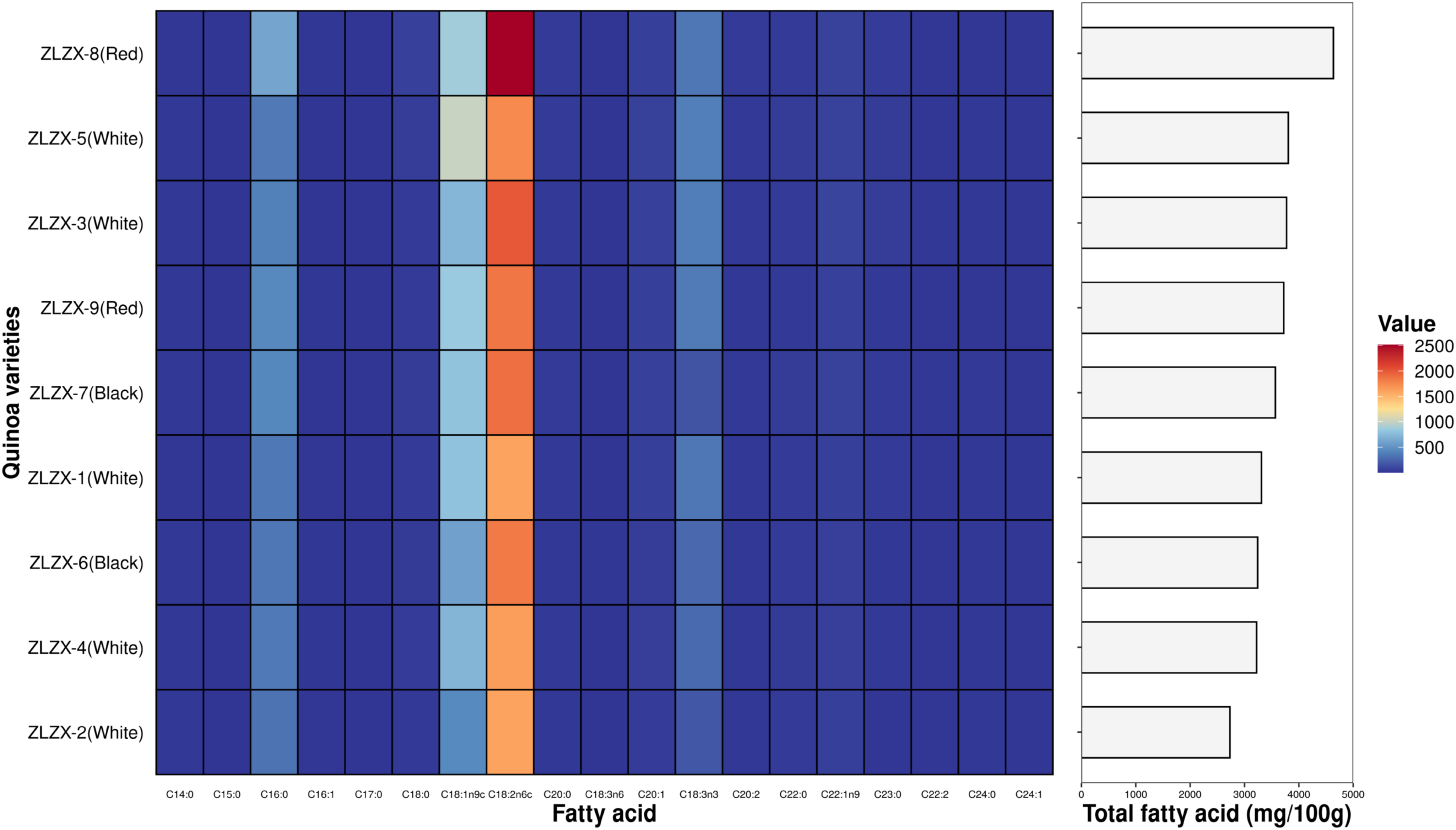
Fatty acids of 9 varieties of quinoa. The heat map on the left shows the content of different fatty acids according to the shade of color, and the histogram on the right shows the total fatty acid content of different types of quinoa.

The fatty acid content was similar to that of Chen et al. (2023). The fatty acid composition of the nine different quinoa varieties is presented in Table S1 and Figure 2. It can be observed from the table that the total content of fatty acids in these nine quinoa varieties ranges from 2732.37 to 4635.62 mg/100 g, with C18:2n6c, C18:1n9c, C16:0, and C18:3n3 being the predominant fatty acids present at levels exceeding 100 mg/100 g. On the other hand, lower amounts of C14:0, C15:0, C16:1, C17:0, C18:3n6, C22:2, and C24:1 were detected. Notably, ZLZX‐8 (red grain) exhibited significantly higher levels of fatty acids compared to other quinoa varieties (*p* < .05). Following this trend were ZLZX‐3 (white grain), ZLZX‐5 (white grain), and ZLZX‐9 (red grain), with no significant differences among them, whereas ZLZX‐2 (white grain) displayed the lowest content of fatty acids.

Studies have found that unsaturated fatty acids have good therapeutic value in the prevention of oxidative stress, inflammation, cardiovascular and cerebrovascular diseases, cancer, and osteoporosis (Liu et al., 2023). In our study, the percentage of unsaturated fatty acids in the total fatty acids of nine quinoa varieties ranged from 83.53% to 86.78%, with ZLZX‐5 (white grain) having the highest proportion, while there was no significant difference among the remaining quinoa varieties. The fatty acid composition of quinoa, as reported by Antonio Vega‐Gálvez et al. (2010), predominantly consists of linoleic acid and linolenic acid, which aligns with our findings. The total content of these essential fatty acids in nine different quinoa varieties ranged from 1784.84 to 2848.46 mg/100 g, accounting for 56.17–65.32% of the total fatty acids present. Among the tested varieties, ZLZX‐8 (red grain) exhibited the highest content, while ZLZX‐1 (white grain) showed the lowest content of essential fatty acids. Furthermore, there was a consistent correlation between the levels of essential fatty acids and total fatty acids across all nine quinoa varieties examined in this study.

### 3.3 Amino acid composition of various quinoa varieties

**FIGURE 3:**
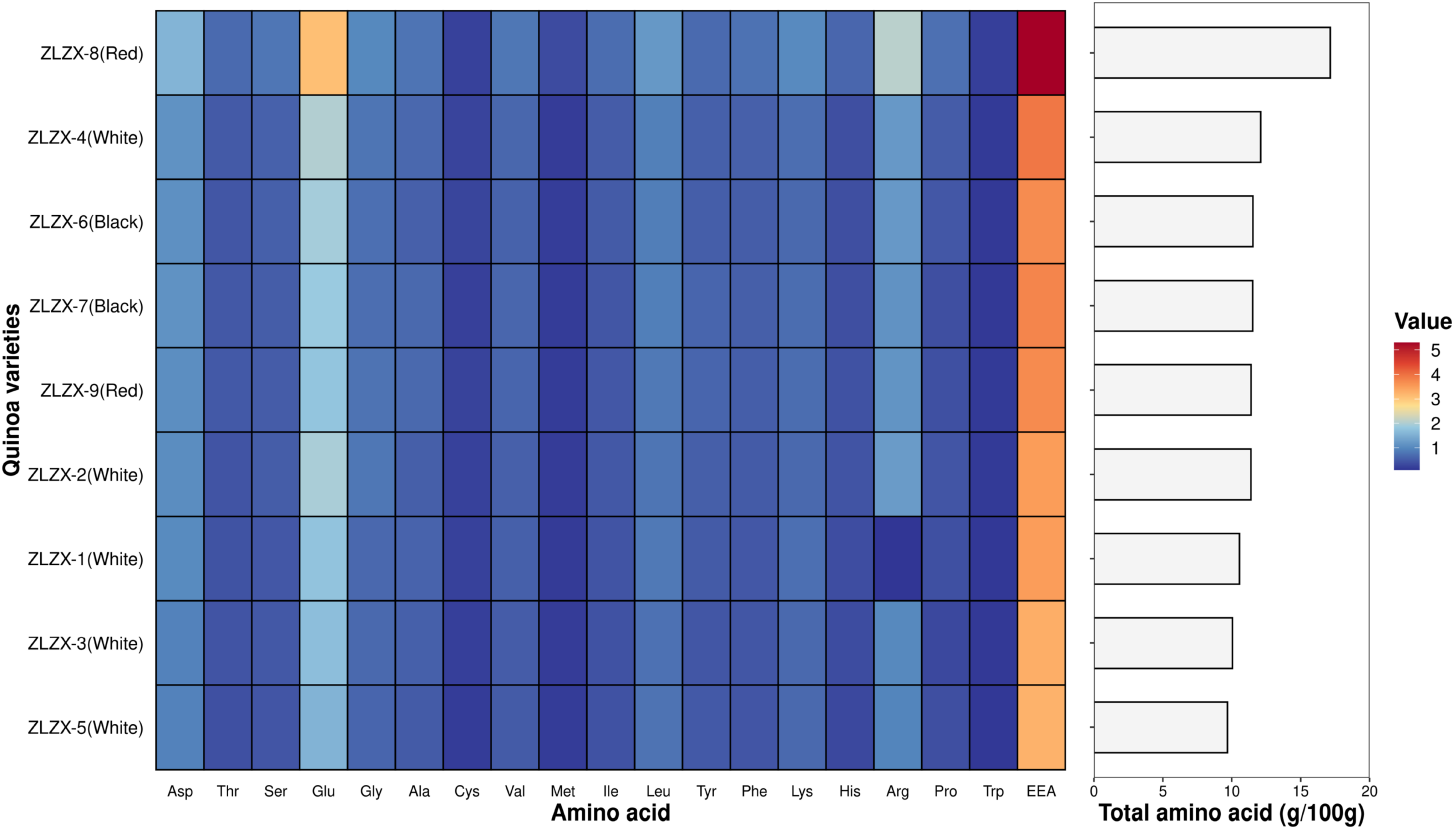
Amino acids of 9 varieties of quinoa. The heat map on the left shows the content of different amino acids according to the shade of color, and the histogram on the right shows the total amino acid content of different types of quinoa.

The amino acid composition of nine different quinoa varieties is presented in Table S2 and Figure 3. It can be observed from the table that all nine quinoa varieties contain a total of 18 essential amino acids. The total amino acid content ranges from 9.69 to 17.15 g/100 g, with aspartic acid, glutamic acid, and arginine being the predominant amino acids present at levels exceeding 1 g/100 g. Additionally, quinoa exhibits a high lysine content, which surpasses that of rice and wheat by more than twofold. However, cystine, methionine, and histidine are found in lower quantities (<0.5 g/100 g). Notably, ZLZX‐8 (red grain) displays the highest total amino acid and essential amino acid contents among all quinoa varieties examined (*p* < .05), significantly surpassing other cultivars such as ZLZX‐4 (white grain), ZLZX‐6 (black grain), and ZLZX‐7 (black grain). Conversely, ZLZX‐5 (white grain) exhibits the lowest content. The total amount of essential amino acids ranged from 3.26 to 5.31 g/100 g, with ZLZX‐8 (red grain) exhibiting the highest content and ZLZX‐5 (white grain) having the lowest content. The total amount of essential amino acids in all nine quinoa varieties was consistent with the overall amino acid levels observed. Furthermore, a comprehensive ranking revealed that red and black quinoa had higher amino acid contents compared to white quinoa, which aligns with the protein content findings mentioned above.

To compare the amino acid composition of nine quinoa varieties and evaluate their amino acid quality, Table 1 presents the amino acid score results. As depicted in the table, a higher score indicates a closer match between the essential amino acid content in quinoa and the recommended ratio, thus indicating a higher protein nutritional value. Among them, methionine + cystine exhibited the lowest amino acid score and chemical score among all nine quinoa varieties, making them the first limiting amino acids. The nine quinoa varieties showed high scores for threonine, leucine, phenylalanine + tyrosine, and lysine, with their contents surpassing FAO recommendations. ZLZX‐8 (red grain), ZLZX‐1 (white grain), and ZLZX‐4 (white grain) had superior essential amino acid scores closest to those of egg protein, whereas ZLZX‐2 (white grain), ZLZX‐7 (black grain), and ZLZX‐9 (red grain) had lower scores.

**TABLE 1:** Amino acid scores in nine varieties of quinoa.

Index	Threonine (mg/g pro)	Valine (mg/g pro)	Methionine + cystine (mg/g pro)	Isoleucine (mg/g pro)	Leucine (mg/g pro)	Phenylalanine + tyrosine (mg/g pro)	Lysine (mg/g pro)
FAO recommended values	28	42	42	42	48	56	42
Egg protein	47	66	57	54	86	93	70
ZLZX‐1 (white)							
Content	30.94	41.83	27.72	32.68	61.76	72.66	54.20
AAS	1.10	1.00	0.66	0.78	1.29	1.30	1.29
CS	0.66	0.63	0.49	0.61	0.72	0.78	0.77
ZLZX‐2 (white)							
Content	28.95	38.15	26.79	32.21	53.61	71.53	44.85
AAS	1.03	0.91	0.64	0.77	1.12	1.28	1.07
CS	0.62	0.58	0.47	0.60	0.62	0.77	0.64
ZLZX‐3 (white)							
Content	32.69	38.58	28.27	30.37	56.58	68.65	49.43
AAS	1.17	0.92	0.67	0.72	1.18	1.23	1.18
CS	0.70	0.58	0.50	0.56	0.66	0.74	0.71
ZLZX‐4 (white)							
Content	34.73	44.68	29.98	36.39	62.86	78.60	50.39
AAS	1.24	1.06	0.71	0.87	1.31	1.40	1.20
CS	0.74	0.68	0.53	0.67	0.73	0.85	0.72
ZLZX‐5 (white)							
Content	29.98	39.77	25.69	31.64	60.43	73.00	51.51
AAS	1.07	0.95	0.61	0.75	1.26	1.30	1.23
CS	0.64	0.60	0.45	0.59	0.70	0.78	0.74
ZLZX‐6 (black)							
Content	31.52	40.97	30.53	34.82	61.10	73.68	45.64
AAS	1.13	0.98	0.73	0.83	1.27	1.32	1.09
CS	0.67	0.62	0.54	0.64	0.71	0.79	0.65
ZLZX‐7 (black)							
Content	33.57	44.44	26.19	32.11	62.83	82.22	49.00
AAS	1.20	1.06	0.62	0.76	1.31	1.47	1.17
CS	0.71	0.67	0.46	0.59	0.73	0.88	0.70
ZLZX‐8 (red)							
Content	37.11	45.33	31.01	38.83	66.85	79.32	56.92
AAS	1.33	1.08	0.74	0.92	1.39	1.42	1.36
CS	0.79	0.69	0.54	0.72	0.78	0.85	0.81
ZLZX‐9 (red)							
Content	33.73	43.15	28.55	32.29	57.73	80.38	48.80
AAS	1.20	1.03	0.68	0.77	1.20	1.44	1.16
CS	0.72	0.65	0.50	0.60	0.67	0.86	0.70

### 3.4 Mineral composition of various quinoa varieties

The mineral composition of nine different varieties of quinoa is presented in Table 2. All nine varieties exhibited high levels of Mg, K, Ca, Mn, Fe, Cu, Zn, and Na. Among the various cultivars, ZLZX‐8 (red grain) displayed significantly elevated concentrations of Mg, K, and Zn compared to other varieties. Similarly, ZLZX‐9 (red grain) demonstrated significantly higher levels of Mn and Fe than the rest. In contrast, ZLZX‐5 (white grain) exhibited notably higher contents of Cu and Na relative to other cultivars. Lastly, ZLZX‐4 (white grain) showcased a significantly greater amount of Ca compared to the remaining varieties. The content of Mn, Fe, Cu, Zn, and Na in quinoa is relatively low. From a health perspective, the high potassium and low sodium characteristics of quinoa align with the recommended intake levels for sodium and potassium in modern nutritional health guidelines. This can effectively contribute to the prevention and reduction of hypertension and cardiovascular diseases, thereby promoting the well‐being of middle‐aged and elderly individuals. Additionally, variations in mineral content among different varieties of quinoa may be attributed to factors such as variety type, maturity stage, light exposure, temperature conditions, and soil composition, among others. Notably, though, there were no significant differences observed in mineral content across various grain colors.

**TABLE 2:** Mineral element content analysis of nine varieties of quinoa (mg/100 g).

Mineral elements	ZLZX‐1 (white)	ZLZX‐2 (white)	ZLZX‐3 (white)	ZLZX‐4 (white)	ZLZX‐5 (white)	ZLZX‐6 (black)	ZLZX‐7 (black)	ZLZX‐8 (red)	ZLZX‐9 (red)
Mg	33.11 ± 0.28 g	137.99 ± 7.72d	196.98 ± 4.61a	179.28 ± 7.48b	155.55 ± 16.60c	52.06 ± 1.08f	178.56 ± 2.87b	200.77 ± 9.66a	106.43 ± 5.08e
K	184.93 ± 3.59f	947.20 ± 21.35c	1202.61 ± 14.02b	1218.56 ± 49.01ab	1260.43 ± 45.44ab	343.01 ± 5.39e	859.61 ± 14.73d	1277.75 ± 40.38a	953.22 ± 13.32c
Ca	39.08 ± 0.52f	83.03 ± 1.57d	126.51 ± 6.63b	181.14 ± 6.87a	86.5 ± 4.66d	47.27 ± 1.50e	102.43 ± 4.30c	110.17 ± 10.18c	140.06 ± 10.09b
Mn	0.417 ± 0.007e	1.90 ± 0.11d	5.05 ± 0.34c	5.05 ± 0.32c	1.75 ± 0.03d	0.61 ± 0.02e	5.78 ± 0.51ab	5.43 ± 0.34bc	6.12 ± 0.29a
Fe	1.21 ± 0.12f	7.51 ± 0.27de	12.38 ± 0.64c	6.62 ± 0.42e	8.36 ± 0.32d	1.22 ± 0.05f	14.67 ± 0.63b	15.62 ± 0.61ab	16.81 ± 1.58a
Cu	0.10 ± 0.003d	1.32 ± 0.06a	0.50 ± 0.09b	0.40 ± 0.009bc	0.42 ± 0.01bc	0.35 ± 0.02b	0.34 ± 0.02bc	1.24 ± 0.16a	0.34 ± 0.009c
Zn	0.34 ± 0.02e	2.65 ± 0.19b	2.87 ± 0.25b	2.17 ± 0.20c	1.86 ± 0.05 cd	0.55 ± 0.02e	1.60 ± 0.11d	4.53 ± 0.29a	1.78 ± 0.07d
Na	2.45 ± 0.19b	1.32 ± 0.03d	0.98 ± 0.06e	1.17 ± 0.03de	6.00 ± 0.23a	0.33 ± 0.01 g	0.52 ± 0.02 fg	1.58 ± 0.02c	0.67 ± 0.04f

*Note*: Different lowercase letters are indicated for comparison at the 5% significant level.

### 3.5 Inositol content of various quinoa varieties

**FIGURE 4:**
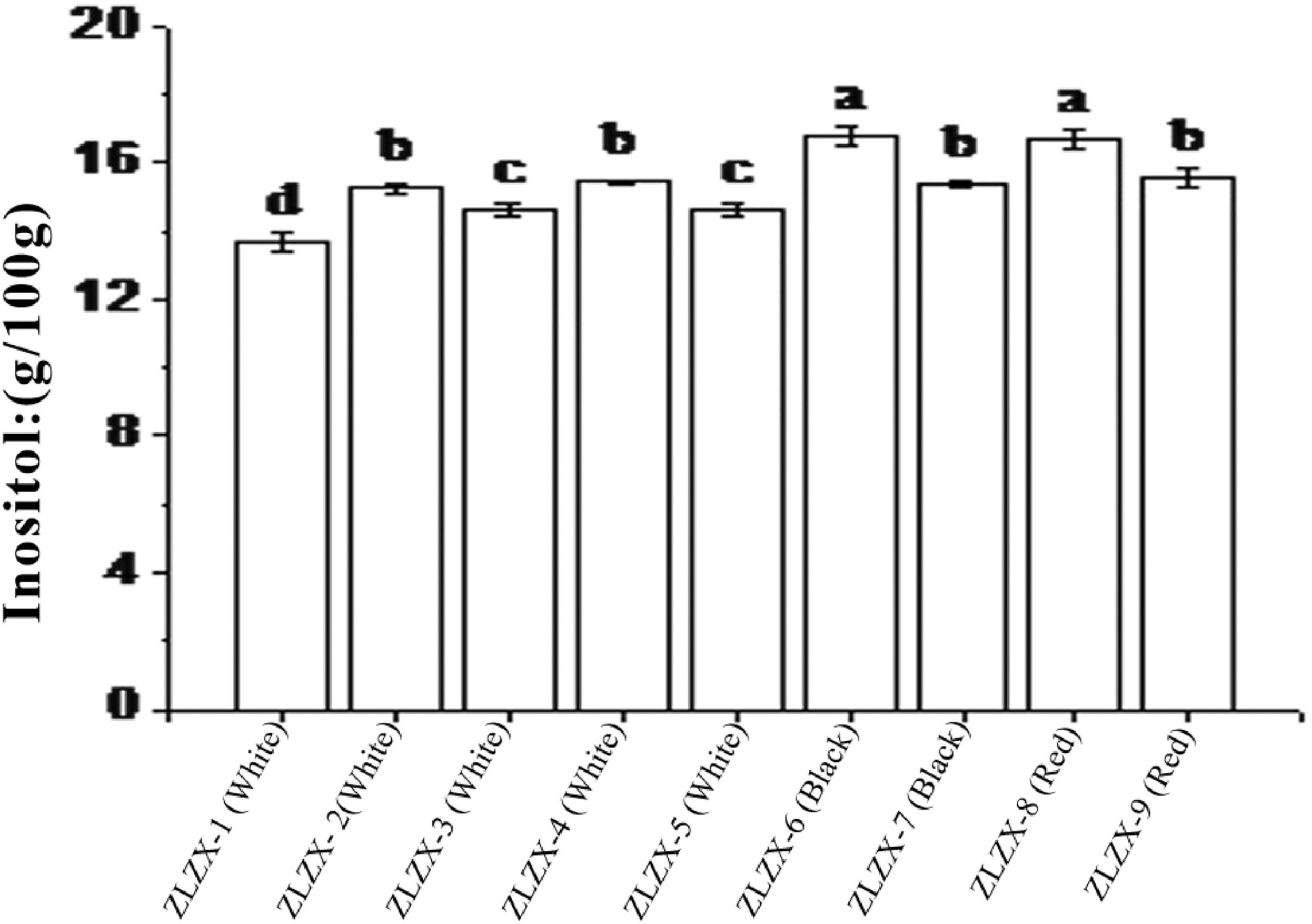
Content of inositol in 9 varieties of quinoa. Different lowercase letters indicate that it is performed at a significant level of 5%.

Inositol is a growth factor for animals and microbes, exhibiting effects similar to those of vitamin B1 and biotin. It plays a crucial role in promoting fat metabolism, reducing cholesterol and triglyceride levels in the body, as well as preventing and treating diseases such as fatty liver, cirrhosis, diabetes, and immunization (Chakraborty et al., 2011). The inositol content of 9 different varieties is presented in Figure 4. It can be observed that ZLZX‐6 (black granule) and ZLZX‐8 (red granule) exhibited the highest levels of inositol, with values reaching 16.76 mg/100 g and 16.67 mg/100 g, respectively. There was no significant difference in the inositol content between these two varieties. Following closely were ZLZX‐2 (white granule), ZLZX‐4 (white granule), ZLZX‐7 (black granule), and ZLZX‐9 (red granule), which showed comparable levels of inositol without any significant differences among them. On the other hand, the lowest content of inositol was found in ZLZX‐1 (leukogranule), as low as 13.68 mg/100 g. Overall, based on a comprehensive ranking analysis of quinoa's inositol content, it can be concluded that white quinoa has lower levels of this compound compared to red and black quinoa.

### 3.6 Polyphenol content and antioxidant activity

The polyphenol content of 9 different varieties is presented in Table 3. Among them, isoxonicin and p‐coumaric acid were identified as the predominant phenolic components in quinoa, with concentrations exceeding 100 mg/kg, while caffeic acid and quercetin exhibited lower levels, below 30 mg/kg. The cumulative amount of six phenolic compounds ranged from 544.97 to 898.9 mg/kg. Notably, ZLZX‐7 (black grain), ZLZX‐8 (red grain), ZLZX‐5 (white grain), and HR‐3 (black grain) displayed higher total amounts compared to other varieties; however, no significant differences were observed among them. Conversely, ZLZX‐3 (white grain) and ZLZX‐1 (white grain) exhibited the lowest total amounts of phenolic compounds. Furthermore, the total amount of phenolic compounds in ZLZX‐7 (black grain) was found to be approximately 1.6 times greater than that in ZLZX‐1 (white grain). Although different quinoa varieties possessed distinct polyphenolic profiles, their composition ratios remained similar; notably, red and black quinoa demonstrated significantly higher polyphenol contents compared to white quinoa.

**TABLE 3:** Phenolic compounds and antioxidant activities of nine varieties of quinoa.

Phenolic components	ZLZX‐1 (white)	ZLZX‐2 (white)	ZLZX‐3 (white)	ZLZX‐4 (white)	ZLZX‐5 (white)	ZLZX‐6 (black)	ZLZX‐7 (black)	ZLZX‐8 (red)	ZLZX‐9 (red)
Isoorientin (mg/100 g)	28.06 ± 0.639e	44.50 ± 1.094c	37.19 ± 0.428d	49.54 ± 1.18b	48.83 ± 1.71b	54.36 ± 1.355a	50.88 ± 1.473b	54.44 ± 1.444a	52.93 ± 3.109b
Caffeic acid (mg/100 g)	0.62 ± 0.009f	1.00 ± 0.005e	0.65 ± 0.027f	1.71 ± 0.025c	2.16 ± 0.04b	1.63 ± 0.039c	1.40 ± 0.024d	0.99 ± 0.031e	2.92 ± 0.117a
Rutin (mg/100 g)	4.97 ± 0.082b	4.08 ± 0.209c	4.72 ± 0.148b	3.27 ± 0.10d	3.57 ± 0.057d	5.05 ± 0.306ab	5.54 ± 0.301ab	5.05 ± 0.33a	4.72 ± 0.271b
p‐Coumaric acid (mg/100 g)	15.89 ± 0.813e	19.11 ± 0.958d	15.75 ± 0.708e	24.58 ± 1.135bc	28.83 ± 0.757a	18.06 ± 1.038de	26.23 ± 1.945b	22.89 ± 1.495c	22.95 ± 0.727c
Ferulic acid (mg/100 g)	4.50 ± 0.148 cd	5.00 ± 0.242abc	5.56 ± 0.185a	4.02 ± 0.215d	4.54 ± 0.207 cd	4.84 ± 0.293bc	5.36 ± 0.326ab	5.35 ± 0.447ab	4.94 ± 0.298abc
Quercetin (mg/100 g)	0.46 ± 0.041 cd	0.41 ± 0.02de	.48 ± 0.007 cd	0.67 ± 0.04b	0.53 ± 0.01c	0.37 ± 0.029ef	0.47 ± 0.028 cd	0.32 ± 0.022f	0.90 ± 0.057a
Total (mg/100 g)	54.50 ± 1.55e	74.09 ± 2.101c	64.35 ± 1.079d	83.80 ± 2.504b	88.45 ± 0.772a	85.80 ± 0.861ab	89.90 ± 1.095a	89.06 ± 3.663a	86.86 ± 0.929ab
ABTS+• (μmol Trolox/gDW)	9.66 ± 0.46d	11.58 ± 0.13b	10.56 ± 0.59c	13.68 ± 0.27a	13.77 ± 0.26a	13.64 ± 0.21a	13.78 ± 0.13a	14.21 ± 0.12a	14.05 ± 0.09a
DPPH• (μmol Trolox/gDW)	12.71 ± 0.34e	16.48 ± 0.52c	14.47 ± 0.12d	17.94 ± 0.11b	18.31 ± 0.11ab	18.32 ± 0.06ab	18.2 ± 0.13ab	18.61 ± 0.08a	18.5 ± 0.06a

*Note*: Different lowercase letters are indicated for comparison at the 5% significant level.

The determination of ABTS+• and DPPH radical scavenging rates can serve as indicators for assessing the antioxidant activity of total polyphenols in quinoa. It is evident from Table 3 that the ABTS+ and DPPH radical scavenging results of nine varieties of quinoa total polyphenols were largely consistent, with ZLZX‐8 (red grain), HR‐3 (black grain), ZLZX‐7 (black grain), and ZLZX‐5 (white grain) exhibiting higher and significantly superior ABTS+ and DPPH free radical scavenging rates compared to other varieties. Conversely, ZLZX‐3 (white grain) and ZLZX‐1 (white grain) displayed the lowest ABTS+ and DPPH radical scavenging rates. The observed trends in ABTS+ and DPPH radical scavenging rates among the nine quinoa species corresponded to the levels of six polyphenolic compounds present, indicating a positive correlation between the higher content of these compounds and increased scavenging rates against both ABTS+• and DPPH radicals.

Based on the analysis of quinoa varieties, ZLZX‐8 (red grain) exhibits a high protein content, essential amino acids, fat, unsaturated fatty acids, exceptional antioxidant activity, as well as elevated levels of potassium and low sodium. These attributes make it particularly suitable for the production of nutritionally fortified foods targeted at elderly individuals and infants. Conversely, ZLZX‐7 (black granule) demonstrates significant dietary fiber and phenolic compound content, making it more appropriate for the development of meal replacement products aimed at obese individuals. Lastly, ZLZX‐5 (white grain) possesses the highest starch content but lower protein levels compared to other varieties. It is rich in quinoa carbohydrates and promotes satiety effectively; therefore, it is best suited as a staple food or when combined with rice, flour, or corn in traditional diets. Other quinoa varieties exhibit minimal differences in nutrient composition and antioxidant activity while maintaining similar quality standards; thus, they can be utilized as raw materials for general quinoa‐based food processing and production.

### 3.7 Principal component analysis

**FIGURE 5:**
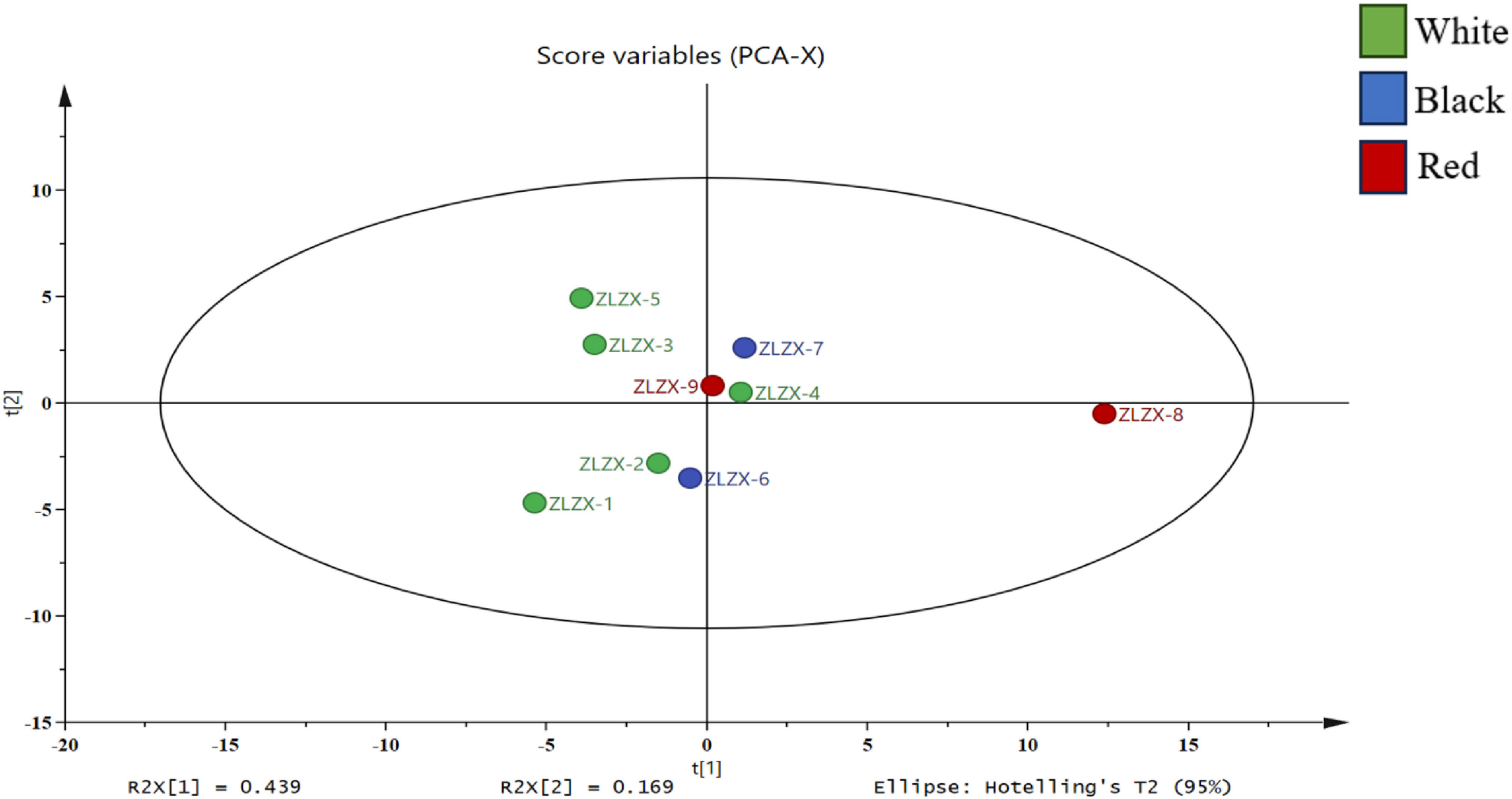
Principal component analysis.

In order to investigate the laws and variations of 61 components in 9 different varieties of quinoa, including nutrients, mineral elements, inositol, phenolic components, and antioxidant activity, SIMCA‐P 11.0 software was employed for data standardization and principal component analysis (PCA). Figure 5 illustrates the overall distribution trend of samples across groups. The first principal component accounted for 43.9% of the total variation, while the second principal component contributed 16.9%, resulting in a cumulative contribution rate of 60.8%. This comprehensive coverage effectively encapsulates sample information. Notably, discernible differences exist in nutritional content among various quinoa varieties; however, color does not significantly correlate with the quality of the quinoa flour. The pigment composition of quinoa with different colors will be further investigated in future studies.

## 4 CONCLUSION

The nutritional components of different quinoa varieties in China were systematically analyzed and compared in this study, highlighting the potential benefits of quinoa as a food source. It was observed that the genetic characteristics significantly influenced the nutritional quality of quinoa, emphasizing the importance of selecting appropriate raw materials based on processing requirements. Moreover, colored varieties of quinoa exhibited superior levels of dietary fiber, inositol, phenolic compounds, ABTS+• scavenging activity, and DPPH free radical scavenging rate compared to white quinoa. However, no significant differences were observed in terms of protein content, amino acid composition, mineral element concentration, and inositol content. Future studies should consider expanding sample sizes to include various colors of quinoa grains and exploring variations in other nutritional components. Additionally, the inclusion of “dark nutrition,” such as miRNA, should be considered. Overall, this research provides valuable insights for selecting raw materials for processing quinoa as well as data support for grading different types of this crop.

## AUTHOR CONTRIBUTIONS

**Lian‐Xin Peng:** Funding acquisition (equal); project administration (equal); resources (equal); supervision (equal); writing – review and editing (equal). **Yuan‐Mou Tang:** Funding acquisition (equal); investigation (equal); supervision (equal). **Yi‐Zhi Liu:** Methodology (equal); visualization (equal); writing – original draft (equal). **Yan‐Hong Zhang:** Conceptualization (equal); data curation (equal); formal analysis (equal); investigation (equal); software (equal). **Ya‐Nan Cao:** Resources (equal); supervision (equal). **Pan‐Pan Song:** Methodology (equal); validation (equal). **Li‐Ming Hou:** Conceptualization (equal); funding acquisition (equal); resources (equal).

## FUNDING INFORMATION

This research was financially supported by the project of Science & Technology Department of Sichuan Province (2023ZYD0078, 2023YFN0062), the Project of the Ministry of Science and Technology of People's Republic of China (2018YFC1602101) and the project of Aba Teachers University (AS‐PYZD2023‐02).

## CONFLICT OF INTEREST STATEMENT

The authors declare no conflicts of interest.

## Supporting information


Table S1.
Table S2.

## Data Availability

The data that support the findings of this study are available in the Tables S1 and S2 of this article.
