# Supplementary material for: A comparative analysis of the nutrient and phytochemical richness among different varieties of quinoa in China
Source: Food Sci Nutr. 2024 Apr 24;12(6):4473–85. doi: 10.1002/fsn3.4113 (PMC11167139; doi:10.1002/fsn3.4113)
Supplement: Supplementary file 1 — Table S1. Table S2. [file FSN3-12-4473-s001.docx]

**Table S1 Contents of fatty acids in 9 varieties of quinoa (mg/100g)**

| Fatty acids | ZLZX-1(White) | ZLZX-2(White) | ZLZX-3(White) | ZLZX-4(White) | ZLZX-5(White) | ZLZX-6(Black) | ZLZX-7(Black) | ZLZX-8(Red) | ZLZX-9(Red) |
| --- | --- | --- | --- | --- | --- | --- | --- | --- | --- |
| C14:0 | 7.7±0.04^f^ | 10.96±0.13^a^ | 10.58±0.16^a^ | 9.76±0.16^bc^ | 9.15±0.22^d^ | 10.09±0.28^b^ | 9.50±0.21^cd^ | 8.3±0.35^e^ | 9.55±0.15^cd^ |
| C15:0 | 2.44±0.09^de^ | 3.18±0.05^ab^ | 2.80±0.05^de^ | 2.99±0.06^cd^ | 2.27±0.2^e^ | 3.27±0.19^ab^ | 3.18±0.10^ab^ | 3.53±0.21^a^ | 2.47±0.24^de^ |
| C16:0 | 345.87±3.92^d^ | 316.44±8.99^e^ | 391.32±6.49^c^ | 353.84±6.36^d^ | 351.61±8.25^d^ | 336.55±18.55^de^ | 423.69±15.42^b^ | 614.49±4.95^a^ | 434.59±0.06^b^ |
| C16:1 | 6.72±0.19^b^ | 8.76±0.16^a^ | 2.88±0.16^f^ | 4.09±0.02^d^ | 3.21±0.13^e^ | 3.78±0.16^d^ | 3.12±0.04^ef^ | 5.25±0.11^c^ | 3.87±0.18^d^ |
| C17:0 | 1.60±0.02^d^ | 1.69±0.04^cd^ | 1.56±0.07^d^ | 2.21±0.12^b^ | 2.06±0.08^bc^ | 1.90±0.04^bcd^ | 2.23±0.17^b^ | 3.57±0.46^a^ | 1.90±0.12^bcd^ |
| C18:0 | 24.05±0.12^d^ | 16.14±0.31^f^ | 21.57±0.35^e^ | 28.63±0.66^d^ | 37.74±1.7^b^ | 25.18±0.3^d^ | 31.5±1.48^c^ | 54.48±1.41^a^ | 21.32±0.71^e^ |
| C18:1n9c | 790.95±8.42^d^ | 437.24±4.68^g^ | 719.34±6.44^e^ | 711.86±3.46^e^ | 1010.44±18.45^a^ | 582.46±8.32^f^ | 797.52±19.09^d^ | 872.64±6.36^b^ | 846.13±9.9^c^ |
| C18:2n6c | 1608.97±15.38^e^ | 1608.97±15.36^e^ | 2006.81±17.32^b^ | 1640.66±41.62^e^ | 1753.79±35.52^d^ | 1839.24±10.19^c^ | 1891.78±45.96^c^ | 2514.56±36.06^a^ | 1856.96±26.87^c^ |
| C20:0 | 16.1±0.12^e^ | 10.41±0.15^h^ | 14.4±0.23^f^ | 17.43±0.31^d^ | 23.99±0.26^a^ | 12.85±0.1^g^ | 21.36±0.71^c^ | 22.6±0.54^b^ | 14.48±0.51^f^ |
| C18:3n6 | 1.27±0.06^de^ | 1.49±0.02^d^ | 1.12±0.02^e^ | 1.15±0.07^e^ | 2.45±0.21^b^ | 5.21±0.05^a^ | 1.88±0.14^c^ | 1.81±0.02^c^ | 1.29±0.07^de^ |
| C20:1 | 45.67±0.64^de^ | 29.76±0.59^g^ | 53.09±0.19^b^ | 43.0±0.94^e^ | 57.33±1.46^a^ | 38.64±0.4^f^ | 51.4±4.24^bc^ | 48.13±1.41^cd^ | 46.71±1.41^de^ |
| C18:3n3 | 339.22±3.86^bc^ | 174.38±4.5^e^ | 372.64±4.66^a^ | 273.3±11.26^d^ | 381.69±10.82^a^ | 262.07±23.35^d^ | 160.68±2.83^e^ | 332.12±9.9^c^ | 357.94±4.24^ab^ |
| C20:2 | 10.53±0.08^c^ | 11.4±0.43^c^ | 14.24±0.12^b^ | 13.7±0.66^b^ | 14.42±0.02^b^ | 10.62±0.39^c^ | 14.86±0.71^b^ | 20.83±1.41^a^ | 10.26±0.71^c^ |
| C22:0 | 22.87±0.45^de^ | 19.58±0.49^f^ | 24.16±0.72^cd^ | 26.47±1.76^c^ | 31.69±0.64^b^ | 20.96±0.85^ef^ | 34.67±2.12^a^ | 21.49±0.64^ef^ | 19.19±0.71^f^ |
| C22:1n9 | 49.34±1.34^d^ | 47.74±1.07^d^ | 73.58±1.4^a^ | 50.58±1.91^cd^ | 65.55±4.54^b^ | 50.42±2.9^cd^ | 64.36±4.24^b^ | 63.58±0.08^b^ | 55.44±0.09^c^ |
| C23:0 | 20.04±0.22^d^ | 8.66±0.16^g^ | 35.12±0.05^a^ | 15.46±0.48^e^ | 32.43±1.41^b^ | 13.08±0.13^f^ | 21.27±1.41^d^ | 28.25±0.14^c^ | 16.71±0.71^e^ |
| C22:2 | 5.4±0.2^f^ | 8.41±0.07^b^ | 9.41±0.05^a^ | 8.28±0.24^b^ | 7.1±0.29^d^ | 7.41±0.06^cd^ | 9.57±0.1^a^ | 7.75±0.07^c^ | 6.59±0.37^e^ |
| C24:0 | 9.07±0.03^de^ | 9.73±0.2^d^ | 10.68±0.27^c^ | 12.33±0.46^b^ | 12.27±0.49^b^ | 11.98±0.15^b^ | 18.33±0.15^a^ | 6.73±0.08^f^ | 8.69±0.42^e^ |
| C24:1 | 5.29±0.1^d^ | 7.43±0.11^b^ | 8.73±0.13^a^ | 7.09±0.15^bc^ | 6.83±0.64^bc^ | 6.42±0.27^c^ | 7.48±0.12^b^ | 5.51±0.46^d^ | 7.39±0.32^b^ |
| Total fatty acids | 3313.09±24.87^d^ | 2732.37±18.58^e^ | 3774.03±12.56^b^ | 3222.86±62.63^d^ | 3806.02±44.27^b^ | 3242.13±29.4^d^ | 3568.39±62.27^c^ | 4635.62±61.46^a^ | 3721.47±6.97^b^ |
| Proportion of unsaturated fatty acids to total fatty acids (%) | 86.43±0.02^ab^ | 85.48±0.4^c^ | 86.43±0.16^ab^ | 85.44±0.02^c^ | 86.78±0.5^a^ | 86.56±0.51^ab^ | 84.14±0.69^d^ | 83.53±0.07^d^ | 85.79±0.11^bc^ |

Note: Different lowercase letters are indicated for comparison at the 5% significant level

**Table S2 Contents of amino acid in 9 varieties of quinoa (g/100g)**

| Amino acid | ZLZX-1(White) | ZLZX-2(White) | ZLZX-3(White) | ZLZX-4(White) | ZLZX-5(White) | ZLZX-6(Black) | ZLZX-7(Black) | ZLZX-8(Red) | ZLZX-9(Red) |
| --- | --- | --- | --- | --- | --- | --- | --- | --- | --- |
| aspartic acid | 1.02±0.025e | 1.05±0.013de | 0.92±0.01f | 1.11±0.018b | 0.92±0.006f | 1.09±0.002bc | 1.11±0.016b | 1.56±0.022a | 1.06±0.004cd |
| threonine | 0.41±0.006e | 0.42±0.013e | 0.43±0.006de | 0.49±0.001b | 0.38±0.016f | 0.45±0.015cd | 0.47±0.006bc | 0.66±0.008a | 0.48±0.013b |
| serine | 0.46±0.006ef | 0.52±0.022d | 0.47±0.008e | 0.57±0.006b | 0.44±0.006f | 0.55±0.011c | 0.52±0.01d | 0.79±0.006a | 0.51±0.011d |
| glutamic acid | 1.75±0.013e | 1.95±0.036b | 1.69±0.029e | 2.01±0.032b | 1.55±0.054f | 1.92±0.028bc | 1.85±0.023cd | 3.11±0.078a | 1.77±0.016de |
| glycine | 0.64±0.004de | 0.80±0.007b | 0.65±0.022d | 0.78±0.013b | 0.61±0.009e | 0.72±0.008c | 0.76±0.01b | 0.99±0.005a | 0.76±0.036b |
| alanine | 0.59±0.008c | 0.54±0.004d | 0.55±0.006d | 0.64±0.016b | 0.50±0.011e | 0.56±0.005d | 0.65±0.008b | 0.77±0.008a | 0.65±0.005b |
| cystine | 0.22±0.011b | 0.22±0.002b | 0.20±0.005b | 0.25±0.008a | 0.16±0.007c | 0.26±0.011a | 0.21±0.006b | 0.22±0.007b | 0.24±0.012a |
| valine | 0.55±0.008d | 0.56±0.011d | 0.51±0.007e | 0.63±0.011b | 0.50±0.005e | 0.58±0.011c | 0.62±0.015b | 0.80±0.009a | 0.61±0.005b |
| methionine | 0.15±0.011c | 0.17±0.005b | 0.17±0.008b | 0.17±0.006b | 0.16±0.004b | 0.18±0.008b | 0.16±0.006bc | 0.33±0.008a | 0.16±0.004bc |
| isoleucine | 0.43±0.01d | 0.47±0.013c | 0.40±0.001e | 0.51±0.006b | 0.40±0.001e | 0.50±0.009b | 0.45±0.002cd | 0.69±0.01a | 0.46±0.011c |
| leucine | 0.82±0.008c | 0.78±0.015d | 0.74±0.018e | 0.89±0.016b | 0.76±0.008de | 0.87±0.025b | 0.88±0.008b | 1.19±0.008a | 0.82±0.004c |
| tyrosine | 0.50±0.004d | 0.54±0.013c | 0.45±0.01e | 0.55±0.01c | 0.48±0.11d | 0.53±0.006c | 0.61±0.01b | 0.66±0.009a | 0.61±0.017b |
| phenylalanine | 0.46±0.012e | 0.50±0.01d | 0.45±0.02e | 0.56±0.018b | 0.44±0.011e | 0.52±0.007cd | 0.54±0.006c | 0.75±0.004a | 0.53±0.006cd |
| lysine | 0.72±0.006b | 0.65±0.008e | 0.65±0.015e | 0.71±0.01bc | 0.65±0.006e | 0.65±0.012e | 0.69±0.005d | 1.01±0.011a | 0.69±0.004cd |
| histidine | 0.35±0.012d | 0.42±0.004b | 0.34±0.017d | 0.37±0.011c | 0.30±0.004e | 0.37±0.007c | 0.37±0.014c | 0.62±0.014a | 0.40±0.008bc |
| arginine | 0.10±0.013d | 1.23±0.03b | 1.00±0.004d | 1.20±0.005b | 0.95±0.006e | 1.20±0.008b | 1.12±0.003c | 2.07±0.028a | 1.14±0.006c |
| proline | 0.38±0.016d | 0.44±0.009c | 0.29±0.006e | 0.51±0.006b | 0.37±0.005d | 0.46±0.013c | 0.37±0.01d | 0.73±0.011a | 0.38±0.008d |
| tryptophan | 0.12±0.003e | 0.14±0.001bc | 0.13±0.005de | 0.14±0.004b | 0.12±0.001e | 0.13±0.003bc | 0.13±0.002cd | 0.20±0.002a | 0.14±0.006bc |
| essential amino acid | 3.51±0.04e | 3.52±0.023e | 3.31±0.026f | 3.94±0.02b | 3.26±0.006f | 3.71±0.014d | 3.79±0.001c | 5.31±0.024a | 3.72±0.024d |
| total | 10.56±0.032e | 11.39±0.006d | 10.05±0.008f | 12.10±0.021b | 9.69±0.02g | 11.54±0.008c | 11.52±0.007c | 17.15±0.053a | 11.40±0.031d |

Note: Different lowercase letters are indicated for comparison at the 5% significant level
